# Ranula: Current Concept of Pathophysiologic Basis and Surgical Management Options

**DOI:** 10.1007/s00268-017-3901-2

**Published:** 2017-02-13

**Authors:** Daniel Kokong, Augustine Iduh, Ikechukwu Chukwu, Joyce Mugu, Samuel Nuhu, Sule Augustine

**Affiliations:** 1 Oto-Rhinolaryngology, University of Jos, Jos, Nigeria; 2 ORL-Head and Neck Surgery, Jos University Teaching Hospital, Jos, Nigeria; 3 Department of ORL-Head and Neck Surgery, Jos University Teaching Hospital, PMB 2076 Jos, Plateau Nigeria; 4 Jos University Teaching Hospital, PMB 2076 Jos, Nigeria; 5 Department of Anaesthesia, College of Medicine, University of Jos & Jos University Teaching Hospital, Jos, Nigeria; 6 Department of General Surgery, College of Medicine, University of Jos & Jos University Teaching Hospital, Jos, Nigeria

## Abstract

**Background:**

There is no consensus opinion on a definitive surgical management option for ranulas to curtail recurrence, largely from the existing gap in knowledge on the pathophysiologic basis.

**Aim:**

To highlight the current scientific basis of ranula development that informed the preferred surgical approach.

**Design:**

Retrospective cohort study.

**Setting:**

Public Tertiary Academic Health Institution.

**Method:**

A 7-year 7-month study of ranulas surgically managed at our tertiary health institution was undertaken—June 1, 2008–December 31, 2015—from case files retrieved utilising the ICD-10 version 10 standard codes.

**Results:**

Twelve cases, representing 0.4 and 1.2% of all institutional and ENT operations, respectively, were managed for ranulas with a M:F = 1:1. The ages ranged from 5/12 to 39 years, mean = 18.5 years, and the disease was prevalent in the third decade of life. Main presentation in the under-fives was related to airway and feeding compromise, while in adults, cosmetic facial appearance. Ranulas in adults were plunging (*n* = 8, 58.3%), left-sided save one with M:F = 2:1. All were unilateral with R:L = 1:2. Treatment included aspiration (*n* = 2, 16.7%) with 100% recurrence, intra-/extraoral excision of ranula only (*n* = 4, 33.3%) with recurrence rate of 50% (*n* = 2, 16.7%), while marsupialisation in children (*n* = 1, 8.3%) had no recurrence. Similarly, transcervical approach (*n* = 5, 41.7%) with excision of both the ranula/sublingual salivary gland recorded zero recurrence. Recurrence was the main complication (*n* = 4, 33.3%).

**Conclusion:**

With the current knowledge on the pathophysiologic basis, extirpation of both the sublingual salivary gland and the ranula by a specialist surgeon is key for a successful outcome.

## Introduction

The term ranula was derived from the Latin word *rana*, meaning frog and ranula describing a little frog, denoting its resemblance to a bulging frog’s underbelly [1]. *Hippocrates* described ranula as due to chronic inflammation, while *Parè* thought ranula represents descent of the brain and the pituitary matter; and *W*. *Boyd* described ranula as a dilatation of the duct of the submandibular gland [2].

Ranulas are rare mucoceles that occur in the floor of the mouth through the mylohyoid muscle dehiscence located at the anterior 2/3 as observed in 45% of cadavers in a study and usually involve the major salivary glands [3, 4]. Specifically, the ranula originates in the body of the sublingual gland, in the ducts of Rivinus of the sublingual gland, and infrequently from the minor salivary glands at this location [5].

The major salivary glands have a unique predilection for developing specific disease patterns: while the parotid gland is the seat for pleomorphic adenomas, the submandibular is for sialolithiasis and the sublingual ranulas [6].

Management of ranulas is a polarising topic, with conflicting evidence as to which treatment modality is best due to the existing gap in knowledge on the current concept of its aetiopathogenesis. A variety of surgical procedures have been quoted in the literature ranging from simple aspiration to complete or partial excision of the ranula and/or the sublingual salivary gland, at times involving the submandibular salivary gland. They include: marsupialisation, dissection, cryotherapy, sclerotherapy, hydro-dissection and LASER ablation. The recurrence rate varies according to the procedure performed [7].

This study was therefore designed to highlight the current concept on the pathophysiologic basis of ranulas/mucocoeles which invariably would influence the choice of an appropriate surgical technique.

## Materials and methods

A 7-year 7-month study of all diagnosed and surgically managed cases of ranulas at the Jos University Teaching Hospital, Nigeria, was undertaken between June 1, 2008, and December 31, 2015. Retrieval of case files utilised the standard codes as contained in the ICD-10 version 10 from the health record’s databank. The medical records were evaluated for the principal demographic, clinical, diagnostic and therapeutic data. We excluded minor operations while generating data. A few pictures taken during an operation depicting the typical ‘frog underbelly’ appearance of ranulas and a typical plunging ranula specimen following surgery were displayed. Diagnosis of ranula was based on clinical presentation, ultrasonographic (USS) findings as confirmed by cytochemical evaluation of the viscous fluid content which yielded mucus and numerous inflammatory cells, the chemical analysis of which showed increased amylase and protein content, suggestive of salivary secretion. However, final confirmation was based on the histopathologic report of a cystic lesion lined by non-keratinising stratified squamous epithelium with a fibrous capsule having central pooling of mucin along with mucinophages following H&E staining. Recurrence was established following the appearance of a cervical swelling at same operative site after a period of at least 6 months which was confirmed as ranula. We followed up our cohort for up to 4 years.

In the data analysis, the frequencies of the variables were generated; simple measures of central tendency and standard deviation were computed, while results were presented in simple descriptive, pictorial forms and figure legends.

The study observed the Declaration of Helsinki.

## Results

**Fig. 1:**
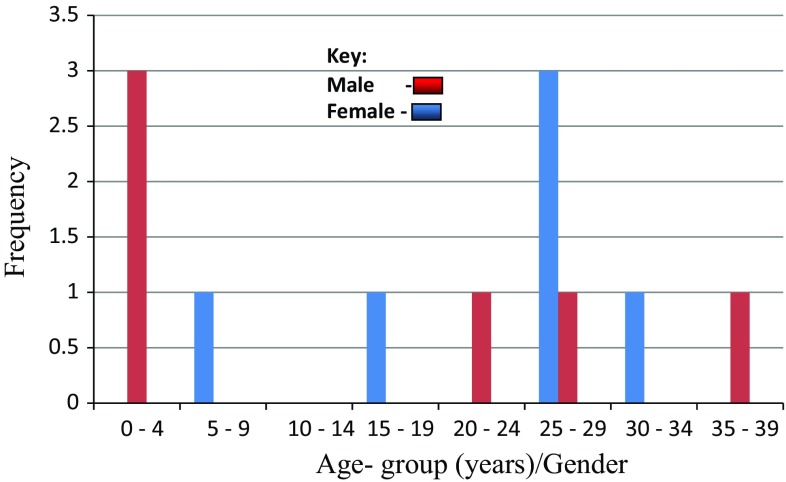
Age–gender distribution of ranulas

Twelve cases were surgically operated on for ranula out of the 2899 institutional operations constituting 0.4% of all operations. Half were performed in ORL—Head & Neck Surgery Department—that operated on 519 patients accounting for 1.2% of all ENT operations. The male-to-female ratio (M:F) was 1:1 with an age range of 5 months–39 years, mean = 18.5 years, median = 25.5 yrs and a mode of 25 years. The disease was prevalent in the third decade of life in the age bracket 25–29 yrs (*n* = 4, 33.3%), closely followed by 0–4 yrs (*n* = 3, 25.0%) (Fig. 1).

Main presentation in the under-five population included: lingual swelling, snoring, obstructive sleep apnoea (OSA), dysphagia, failure to thrive (FTT), and upper airway obstruction (UAO), while in adults, cosmetic facial appearance majorly except for a 25-year-old that had a recurrence 10 years post-excision by a non-specialist surgeon at a different facility who presented with dysphagia, noisy breathing, hot potato speech and impending upper airway obstruction, in addition.

Duration of symptoms varies depending on age and size. In early life, it can be as short as at birth to less than 6 months, while in adults, as long as 10 years. Ranulas in adults were plunging with a male-to-female ratio (M:F) = 2:1 and were unilateral save for one with a right-to-left ratio (R:L ratio) = 1:2.

**Fig. 2:**
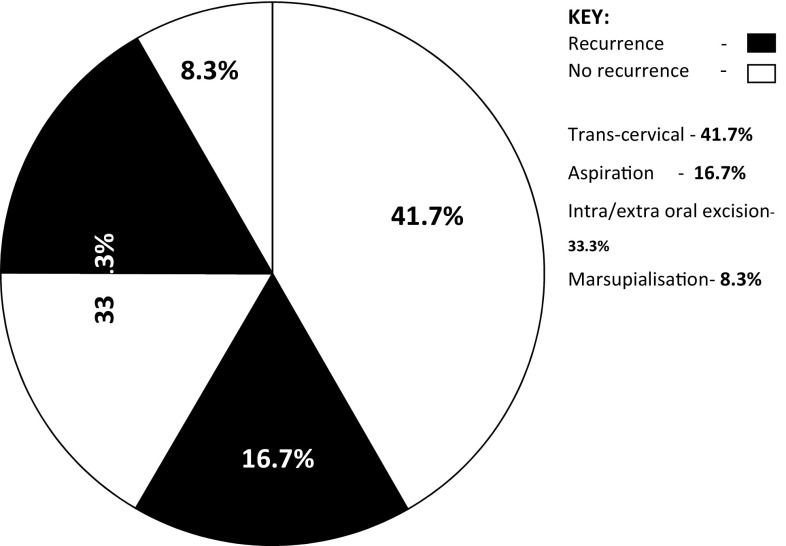
Various surgical treatment modalities offered, with their recurrence rates

**Fig. 3:**
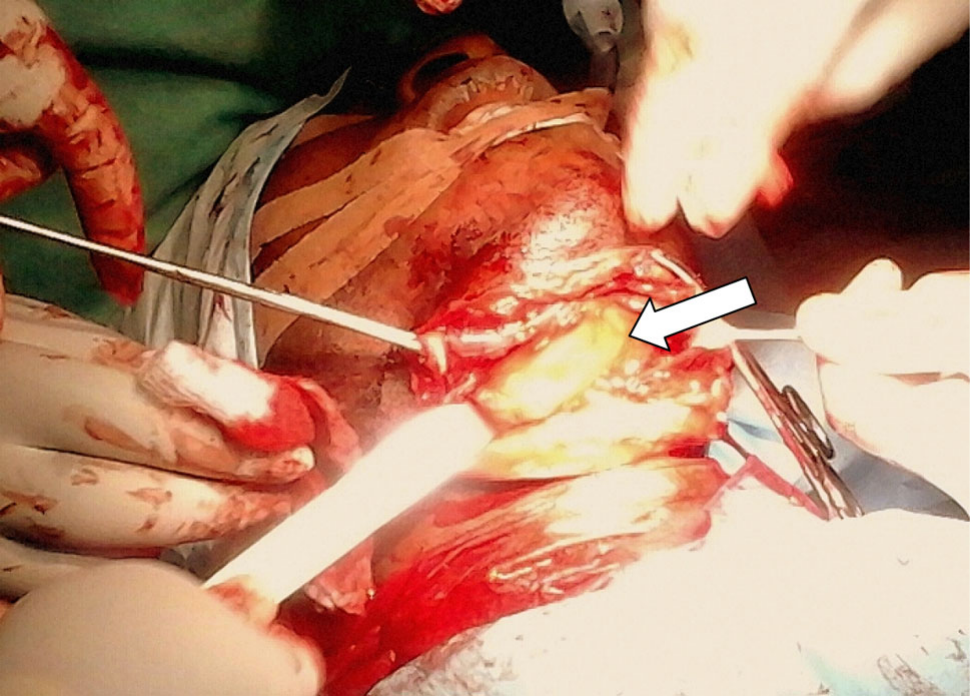
Frog underbelly appearance of ranulas (*arrow*). Picture taken during an operation via a transcervical approach on a 25-year-old male peasant farmer with a right-sided plunging ranula

**Fig. 4:**
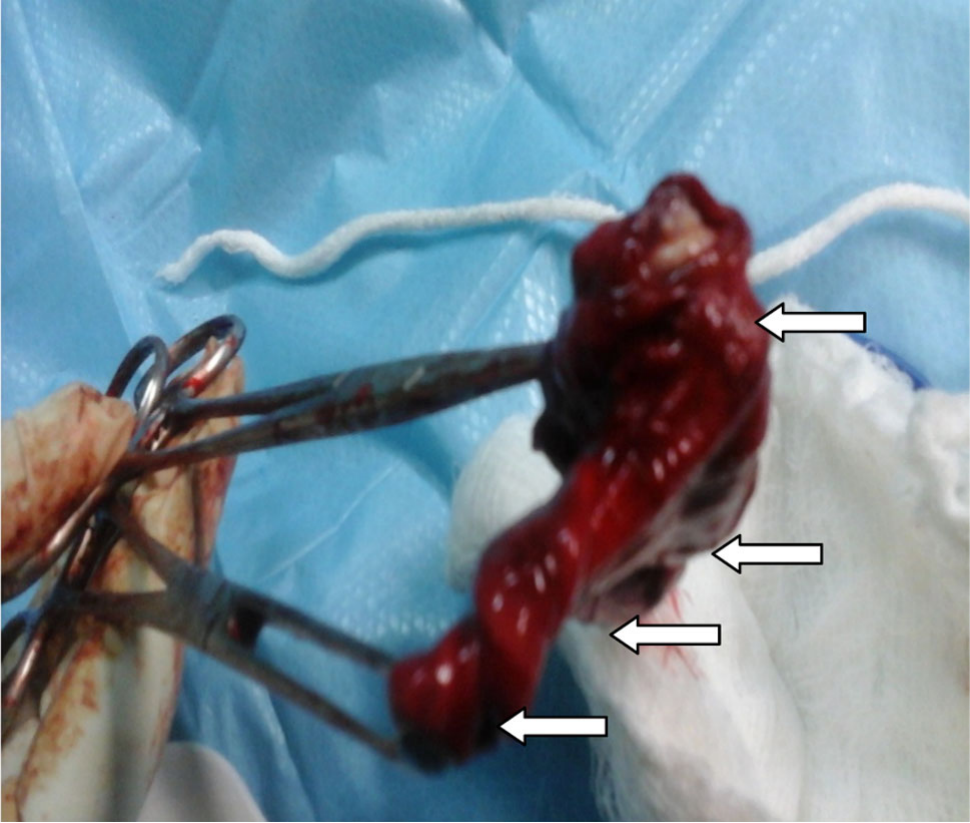
A plunging ranula surgical specimen on completion of the earlier-shown operation. This depicts a typical plunging ranula that consists of the cyst (first *arrow* above) and a ‘tail’ (the tail comprises the neck, stalk and the extirpated sublingual salivary gland)—the ‘tail sign’ phenomenon is pathognomonic (subsequent three *arrows* downwards, respectively)

**Fig. 5:**
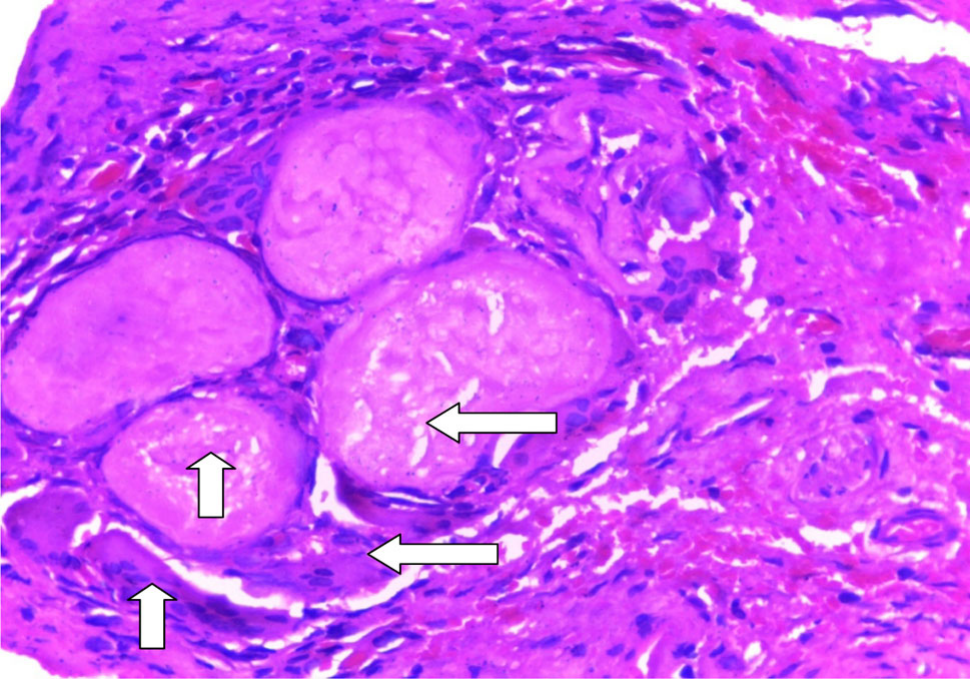
A photomicrograph of the ranula specimen following H&E low power (×10) displaying pools of mucin surrounded by inflammatory cells and fibrosis. Also seen are giant cells (mucinophages) (*arrows*)

**Fig. 6:**
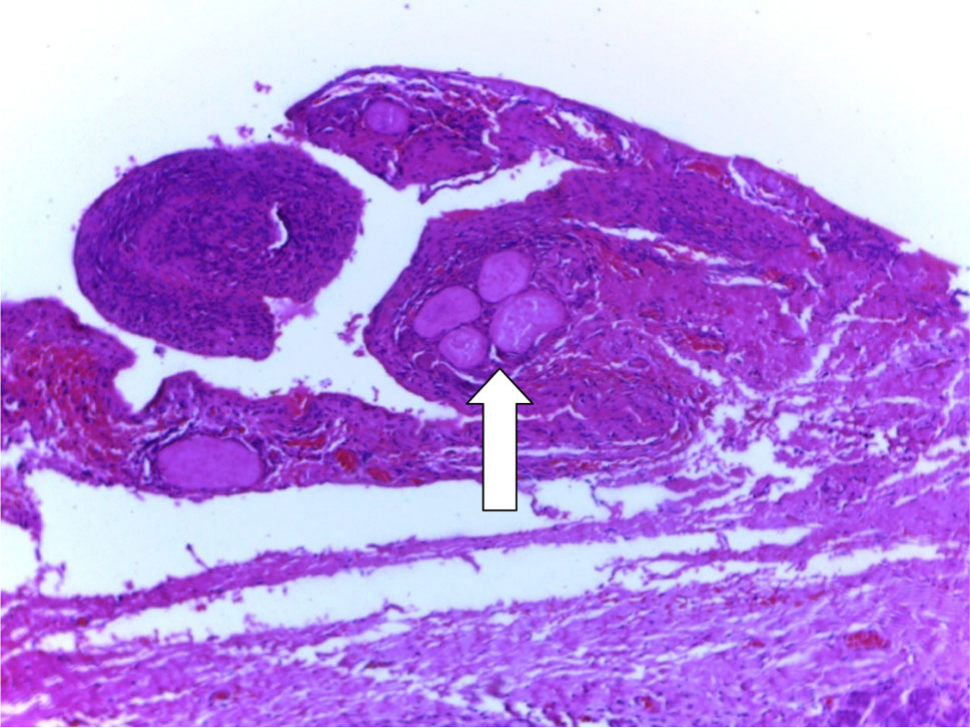
A photomicrograph of the ranula specimen following H&E low power (×10) showing extracellular pools of salivary mucin (*arrow*) surrounded by inflammatory cells and fibrosis

**Fig. 7:**
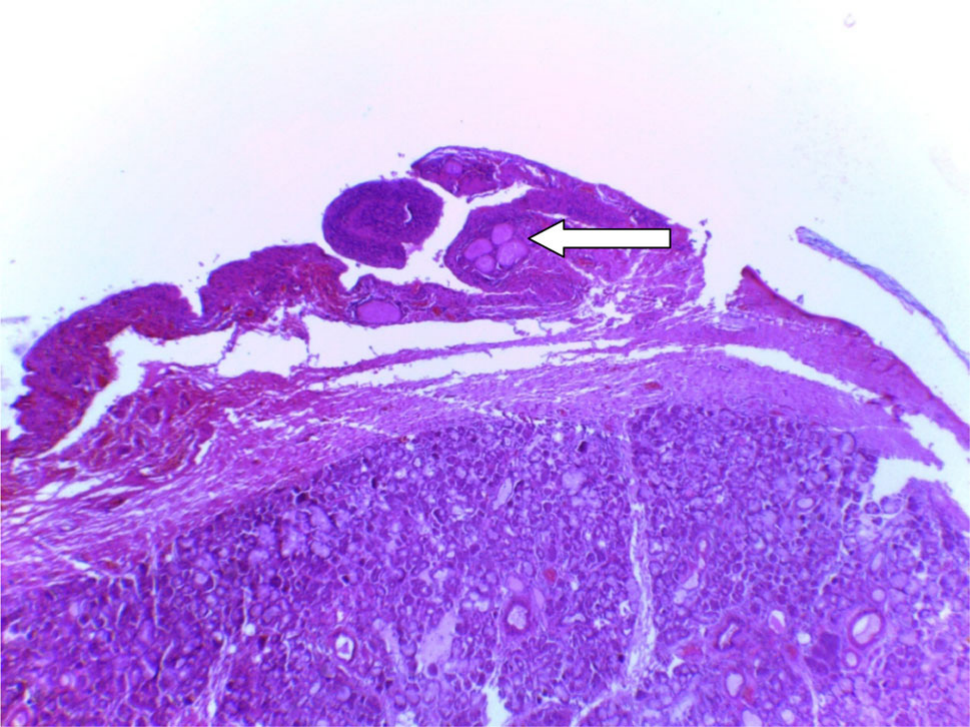
A photomicrograph of the ranula specimen following H&E low power (×4) showing extracellular pools of salivary mucin (*arrow*) surrounded by inflammatory cells and fibrosis. Normal salivary gland tissue seen below confirming our approach of en bloc removal of both ranula and the offending sublingual salivary gland

Treatment included aspiration (*n* = 2, 16.7%), with 100% recurrence, intra-/extraoral excision of ranula only (*n* = 4, 33.3%) with half having recurrence (*n* = 2, 16.7%), while marsupialisation in children (*n* = 1, 8.3%) having no recurrence (Fig. 2). Similarly, transcervical approach (*n* = 5, 41.7%) with blunt dissection for excision of both the ranula and sublingual salivary gland which were plunging ranulas recorded zero recurrence after the follow-up of up to 4 years. (See Figs. 3, 4 for a typical ranula appearance and a typical surgical specimen of a plunging ranula, while Figs. 5,6, 7 are the photomicrographs of the surgical specimen following H&E staining). We utilised the modified inverted hockey stick or apron incisions for our approach depending on the ranula size, shape, orientation and extension.

The main complication observed was recurrence (*n* = 4, 33.3%).

## Discussion

Ranulas are rare surgically amenable diseases of the salivary glands as described by various researchers [8], which was confirmed in this study with a prevalence of 0.4% of institutional operations and 1.2% of ENT operations. In an 18-year retrospective study at the North Carolina University in the USA, only 26 cases of ranulas were reported [7]. We may propose that rarity of the disease may be responsible for the dearth of literature on the subject; more so, several studies by most scholars have been case reports [9]. Studies reported female gender predilection with no clear scientific basis including those of Chidzonga et al. [10] and Zhao et al. [11] though with a distinct predilection for males in cases of plunging ranulas in the latter; this was reversed in this study in a ratio 2:1, but was in agreement with the diving/plunging ranulas.

No age is spared for ranulas. Our series recorded an age range of 5 months–39 years which is at variance with a study which recorded 3–61 years [2].

Ranulas diagnosed on routine antenatal large enough to warrant intrauterine decompression for a safe labour and delivery have been reported [12]. This has suggested a genetic basis for ranulas of early life including plunging ranulas which were found to be more frequent in the ethnic groups of the Maori and the Pacific Island Polynesians [13, 14]. Intra-oral ranulas have been found frequently in early life and young adults [11] which was observed in this study where all the ranulas were seen in the younger age group (*n* = 5, 41.7%). Ranulas develop slowly and typically present in the second and third decades of life [15] or even later in life and are commonly plunging as observed in our series with a male-to-female ratio of 2:1. For unknown reason, the plunging ranulas were reported to have a predilection for the right side [10, 11] which was at variance with our study in which all were left-sided save one. The duration of symptoms tends to be shorter in children and young adults than in the adults. We may speculate that the oral cavity which subserves vital functions of deglutition and respiration can easily be compromised by a space occupying lesion as it is situated in a small space with rigid boundaries which make early presentation the norm. This was observed in our series as all the children presented with failure to thrive (FTT) from compromised feeding, while in compromised airway, presentations were majorly noisy breathing, snoring, obstructive sleep apnoea (OSA) and impending upper airway obstruction with duration of symptoms less than 6 months. In adults, however, the duration of symptoms tends to be longer; this is because the oral cavity is wider as ranulas tend to expand gradually and herniate through the mylohyoid dehiscence, and extend into deep neck spaces to appear in the neck and distant locations, hence the name ‘diving/plunging’. Intra-thoracic extensions of plunging ranulas have been documented in adults [15–17].

There is no consensus opinion on the definitive management of these lesions, and there is often great variation in practice. Multiple options exist, including surveillance, needle aspiration, surgical excision of the cyst, sublingual gland excision along with the cyst, marsupialisation, sclerotherapy, laser excision or cryosurgery [17]. The sclerotherapy employs bleomycin—antineoplastic antibiotic of *Streptomyces verticillus*—OK-432 (Picibanil), a lyophilised mixture of low virulent strain of *Streptococcus pyogenes* incubated with benzyl penicillin, that have been found to produce good effect [18, 19]. In lesions diagnosed antenatally where the oral mass can be life-threatening, Kolker et al. [20] described the ex utero intrapartum treatment (the EXIT technique).

However, different outcomes have been reported with each approach having varying complications. Recurrence has been reported the main culprit with different scholars advocating excision of either ranula alone, ranula with the sublingual gland or ranula with the submandibular gland. This goes to demonstrate that the aetiopathogenesis of ranulas was yet to be fully understood.

The current scientific knowledge reveals that ranulas originate primarily from the sublingual salivary gland which is a spontaneous secretor of saliva, that is, produces saliva without parasympathetic stimulation that occurs during feeding, which is drained by 6–20 ducts scattered in the floor of the oral cavity called ducts of Rivinus. They are located majorly at the posterior and superior aspects, while at the anterior part, they coalesce into a single duct termed the Bartholin’s duct which empties into the Wharton’s duct of the submandibular salivary gland. The sublingual salivary gland is almond shaped, weighs 2–4 g and produces mainly mucus secretions. It lacks a true capsule but rather mucosal fold of the floor of the mouth which envelopes it [21]. The gland is resistant to obstruction because of this unique anatomical arrangement.

Congenitally, ranula occurs following imperforate salivary gland duct and ostial stenosis leading to cyst formation. Trauma to the sublingual gland duct leads to mucus extravasation into the submucosa via hydrostatic pressure and formation of pseudocyst from mucus escape reaction (MER). Trauma directly damages the acini with consequent ductal obstruction, and back-pressure of secretion builds up with subsequent acini rupture. Subsequently, there is increased hydrostatic pressure, extravasation of mucus, and then pseudocyst formation. Congenital narrowing of the duct, dehiscence of the mylohyoid muscle and sialolithiasis have also been implicated in ranula formation. This was confirmed in a study where experimental ligation of sublingual gland duct resulted in ranula formation, while ligation of submandibular gland did not and that of parotid gland led to atrophy [2].

Regarding superficial mucoceles, however, trauma does not always appear to play an important role in the pathogenesis. In many cases, mucosal inflammation that involves the minor gland duct results in blockage, dilatation and rupture of the duct with subepithelial spillage of fluid. Changes in minor salivary gland function and composition of the saliva may contribute to their development. In some cases, an immunological reaction may be the cause. Studies have revealed increased levels of matrix metalloproteinase, tumour necrosis factor-α, type IV collagenase and plasminogen activators in mucoceles compared with that of whole saliva. These factors are further hypothesised to enhance the accumulation of proteolytic enzymes that are responsible for the invasive character of extravasated mucus [22, 23].

In a study, Sigismund et al. [4] in a retrospective analysis of 65 patients reported a recurrence prevalence of 3.6% following complete excision of the sublingual gland alone compared with 36.7% prevalence with ranula excision alone; by implication, the former is ×10 better than the latter. He did not perform combined excision of the ranula with the sublingual salivary gland.

In our series, recurrence following aspiration was 100%, while that by intra-/extraoral ranula excision alone was 50%. These were done mainly by the non-specialist surgeons. However, combined ranula with the sublingual gland excision yielded zero recurrence so was the only case in an infant that had marsupialisation. We utilised the transcervical approach with blunt dissection to approach the ranula and remove the sublingual salivary gland for plunging ranulas rather than combined transcervical with transoral approaches. We employed any of the various neck incision types suitable for a particular case depending on the size, shape, extent and orientation of the ranula. The modified inverted hockey stick or apron incisions would suffice for most presentations.

Ranula is a clinical diagnosis, and imaging studies are done mainly to know the extension of swelling prior to surgery or when the diagnosis is unclear. Computed tomography and specifically the presence of ‘tail sign’ is pathognomonic for the plunging ranula [24–26]. This ‘tail’ is due to extension behind the mylohyoid muscle and confirms the ranula to arise from the sublingual gland [27]. This may explain the zero recurrence in our combined ranula with sublingual salivary gland excision approach which was also observed in a study [7]. We may not advance explanations for the resolution observed in 50% of the cases that had only ranula excision, but the success rate in marsupialisation and micro-marsupialisation in children has been documented [28].

In addition, confirmation would be required via cytochemical analysis to demonstrate the characteristic viscous fluid content laden with mucin, inflammatory cells, protein, salivary amylase indicating salivary gland origin. The cystic lesion is, however, confirmed histopathologically as ranula by the presence of peripheral fibrosis, lined by non-keratinising stratified squamous epithelial layer with central pool of mucin, inflammatory cells and mucinophages following H&E staining [29] as demonstrated in our series.

## Conclusion

With the current knowledge of the pathophysiologic basis, extirpation of both the sublingual salivary gland and the ranula by a specialist surgeon is key for a successful outcome.
